# Inositol polyphosphate multikinase signaling in the regulation of metabolism

**DOI:** 10.1111/j.1749-6632.2012.06725.x

**Published:** 2012-10-10

**Authors:** Joo-Young Lee, Young-ran Kim, Jina Park, Seyun Kim

**Affiliations:** 1 Department of Biological Sciences, Korea Advanced Institute of Science and TechnologyDaejeon, Korea; 2 College of Pharmacy, Chungnam National UniversityDaejeon, Korea

**Keywords:** inositol polyphosphate, IPMK, mTOR, AMPK, growth, metabolism

## Abstract

Inositol phosphates (IPs) act as signaling messengers to regulate various cellular processes such as growth. Inositol polyphosphate multikinase (IPMK) generates inositol tetrakis- and pentakisphosphates (IP_4_ and IP_5_), acting as a key enzyme for inositol polyphosphate biosynthesis. IPMK was initially discovered as an essential subunit of the arginine-sensing transcription complex in budding yeast. In mammals, IPMK is also known as a physiologically important phosphatidylinositol 3 kinase (PI3K) that forms phosphatidylinositol 3,4,5-trisphosphate (PIP_3_), which activates Akt/PKB and stimulates its signaling. Acting in a catalytically independent fashion, IPMK mediates the activation of mammalian target of rapamycin (mTOR) in response to essential amino acids. In addition, IPMK binds and modulates AMP-activated protein kinase (AMPK) signaling pathways, including those involved in hypothalamic control of food intake. These recent findings strongly suggest that IPMK is a versatile player in insulin-, nutrient-, and energy-mediated metabolism signaling networks. Agents that control IPMK functions may provide novel therapeutics in metabolic syndromes such as obesity and diabetes.

## Introduction

Inositol is a naturally occurring glucose isomer and a key nutrient in the human diet.^1^*Myo*-inositol, a six-carbon cyclitol that contains one axial and five equatorial hydroxyl groups, is the major form out of nine possible inositol isomers. Inositol was formerly assumed to be an essential B complex vitamin; however, inositol is now known to be synthesized *de novo* in the human body. For instance, one normal adult kidney can produce a few grams of inositol per day.^2^ Inositol is converted from glucose-6-phosphate (G6P) through two biochemical reactions: G6P is first isomerized by inositol 3-phosphate synthase to inositol 3-phosphate, which is dephosphorylated by inositol monophosphatase 1 to yield free *myo*-inositol. In plants, inositol hexakisphosphate (IP_6_) is known as phytic acid, which is enriched in cereals and is utilized for phosphorus storage. When the levels of inositol become extremely low, severe medical disturbances, such as diabetic changes, anxiety disorders, and hypercholesterolemia ensue.^1^,^3^

**Figure 1:**
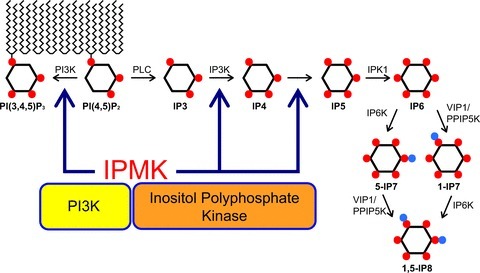
Inositol phosphate biosynthesis in mammals. PIP_2_ is phosphorylated by p85/p110 PI3K or IPMK to produce PIP_3_. Phospholipase C (PLC)-dependent hydrolysis of PIP_2_ produces IP_3_. Phosphorylation of IP_3_ by IP3K or IPMK generates IP_4_ (Ins(1,3,4,5)P4 or Ins(1,4,5,6)P4). For simplicity, Ins(1,3,4,5)P4 is depicted here. Subsequent actions of IPMK and inositol-1,3,4,5,6-pentakisphosphate-2-kinase (IPK1) on IP_4_ ultimately yield IP_6_. IP6Ks phosphorylate the 5 position, generating 5-PP-IP_5_ (5-IP_7_) from IP_6_ and 1,5-(PP)2-IP_4_ (1,5-IP_8_) from 1-PP-IP_5_. Human VIP/diphosphoinositolpentakisphosphate kinase (PPIP5K) phosphorylates the 1/3 position to form 1/3-PP-IP_5_ (labeled 1-IP_7_) from IP_6_ or 1,5-IP_8_ from 5-IP_7_. The multiple enzymatic functions of IPMK in IP biosynthesis are highlighted.

Aside from a structural role for inositol (in the form of phosphatidylinositol) in maintaining the cellular membrane bilayer, inositol is viewed as a secondary signaling messenger that controls diverse physiologic events.^4^–^6^ The best characterized signaling function for inositol polyphosphate, defined as an inositol harboring more than one phosphate, is inositol 1,4,5-trisphosphate (IP_3_)-mediated cytosolic calcium release.^7^,^8^ Upon growth factor stimulation, activated phospholipases hydrolyze phosphatidylinositol 4,5-bisphosphates (PIP_2_) into water-soluble IP_3_ and lipid-anchored diacylglycerol. IP_3_ then binds to the IP_3_ receptor, which is localized on the membranes of intracellular calcium storage sites such as the endoplasmic reticulum, and subsequently elevates cytosolic calcium levels by stimulating the calcium channel activity of the IP_3_ receptor. Since the discovery of IP_3_ and its signaling role in the mid-1980s, continuing investigations of the biochemical fate of IP_3_ inside the cell have revealed the presence of a family of inositol phosphate kinases and associated specific phosphorylation events on the inositol ring at different hydroxyl groups (Fig. 1). Higher inositol polyphosphates include inositol pyrophosphates (e.g., IP_7_), which contain diphosphates with one or more high energy-harboring pyrophosphate bonds. Multiple approaches, including genetic manipulation of inositol polyphosphate metabolism and biochemical characterization of IP-binding signaling targets, have recently highlighted the physiologic significance of inositol poly- and pyrophosphates in the regulation of major physiologic events such as growth and apoptosis (reviewed in Ref. 4). In this review, we will focus on inositol polyphosphate multikinase (IPMK) and its signaling functions in controlling mammalian target of rapamycin (mTOR) and adenosine monophosphate (AMP)-activated protein kinase (AMPK), two major central molecules in metabolism signaling networks.

## IPMK and IP metabolism

In the process of cloning of IP_6_ kinase genes, Snyder and his associates identified IPMK as an enzyme essential for the synthesis of IP_4_ (both Ins(1,3,4,5)P4 and Ins(1,4,5,6)P4) and IP_5_ (Ins(1,3,4,5,6)P5).^9^,^10^ In organisms from yeast to humans, IPMK is often the only enzyme responsible for converting IP_4_ into IP_5_. Thus, IPMK deletion abolishes the formation of IP_5_, but it also eliminates IP_6_ and IP_7_ formation, indicating that IPMK is an essential enzyme in the generation of all highly phosphorylated IP species, including IP_7_.^11^,^12^ Its unique broad substrate specificity also enables IPMK to operate as a lipid-soluble inositol kinase that can phosphorylate PIP_2_ at the 3 position to produce phosphatidylinositol 3,4,5-trisphosphate (PIP_3_) (Fig. 1).^13^,^14^ IPMK deletion in mouse embryonic fibroblast results in a 50% decrease in PIP_3_ levels as well as significantly reduced PIP_3_-dependent Akt signaling in response to growth stimuli such as insulin and epidermal growth factor, suggesting that IPMK functions physiologically as a PI3 kinase.^14^

## Yeast IPMK and arginine metabolism

Possible IPMK functions in mammalian metabolic homeostasis were suggested by previous studies in budding yeast designed to screen for genes that regulate metabolic activities in response to arginine availability.^15^ IPMK was initially cloned as Arg82/ArgRIII, one of the proteins involved in the regulation of arginine metabolism, and is now designated Ipk2 or yeast IPMK.^9^,^10^,^15^ Arg80-Mcm1 is an arginine-sensing transcriptional complex that governs the expression of genes required for arginine metabolism. Yeast IPMK, acting independently of its catalytic activity, is crucial for assembling the Arg80-Mcm1 protein complex at the promoter region of genes involved in arginine metabolism.^16^,^17^ However, the kinase activity of yeast IPMK and production of inositol polyphosphates are also required for the appropriate stimulation of the Arg80-Mcm1 transcription complex, as demonstrated in phenotypic cell growth assays.^10^,^18^ Restoration of the IPMK products, IP_4_ and IP_5_, by overexpressing the IPMK gene from evolutionarily divergent plant or fruit fly functionally rescues the growth defect in IPMK-null yeast cells.^18^,^19^

## IPMK and mTOR signaling

Among the many factors that contribute to the regulation of anabolic pathways (e.g., protein synthesis) in mammals by growth factors and nutrients such as essential amino acids, the mTOR complex 1 (mTORC1) is considered to be a central signaling regulator (reviewed in Ref. 20). mTOR is a serine/threonine protein kinase that exists as two distinct protein complexes; mTORC1 is defined by an association of mTOR with the binding proteins raptor, PRAS40, DEPTOR, and mLST8, whereas the integrity of mTORC2 depends on rictor, Protor, mSIN1, DEPTOR, and mLST8. The rapamycin-sensitive mTORC1 complex promotes anabolic reactions such as protein synthesis and inhibits autophagy, whereas the rapamycin-insensitive mTORC2 complex exerts discrete functions as an Akt serine 473 kinase. Signaling pathways by which insulin and other growth factors stimulate mTORC1 have been well delineated. Growth factors act through PIP_3_ to activate the PI3K pathway, which stimulates protein kinase Akt/PKB. Active Akt, in turn, phosphorylates TSC2, abrogating TSC2-mediated inhibition of Rheb GTPase, which can directly stimulate mTORC1. The entire process by which nutrient amino acids activate mTORC1 has remained elusive. However, a growing body of evidence clearly suggests that in response to amino acids, Rag GTPases, in association with the Ragulator complex, mediates the spatial redistribution of inactive mTORC1 to the surface of lysosomes, which contain mTOR-activating Rheb GTPase.^21^–^23^ How these molecular events are controlled to promote mTORC1 activation and protein translation is largely unknown, although the vacuolar H(+)-adenosine triphosphatase ATPase and leucyl-tRNA synthetase have been recently discovered as essential factors for amino-acid sensing.^24^,^25^

In searching for roles of IPMK in mammalian metabolic signaling systems, Kim *et al.* identified IPMK as a novel mTOR cofactor and a critical determinant of amino acid-induced mTORC1 signaling.^12^ Deletion of IPMK in mouse embryonic fibroblasts markedly reduces both amino acid-stimulated mTORC1 activation and leucine-induced cell growth, effects comparable to the deletion of raptor, an essential subunit of mTORC1-signaling responses to essential amino acids. Interestingly, a catalytically incompetent IPMK mutant stimulates mTORC1 as effectively as wild-type IPMK.

The fact that selective and significant disruption of mTOR binding to raptor does not produce a defect in mTORC2 stability led to the discovery of IPMK as a novel cofactor for mTORC1 signaling. Mammalian IPMK genes contain a unique amino terminal domain indispensable for the regulation of IPMK-dependent mTORC1-signaling responses to amino acids. This mammalian-specific amino terminus of IPMK mediates direct binding of IPMK with mTOR. Overexpression of the amino terminal 60 amino acids of human IPMK in HEK293 cells selectively interrupts endogenous mTOR-raptor interactions in the presence of amino acids and inhibits amino acid-induced mTORC1 signaling. Taken together, these observations suggest that IPMK is a physiologic cofactor of mTOR, maintaining mTORC1 stability and thereby mediating the ability of amino acids to fully activate mTORC1. Presumably, IPMK has evolved from a scaffolding protein that assembles the arginine sensor in budding yeast to a cytosolic, mTOR-associated factor that mediates the stable interaction between mTOR and raptor in mammalian cells.^12^,^26^

## IPMK and AMPK signaling

AMPK is an evolutionarily conserved energy sensor that triggers catabolic energy fluxes and antagonizes ATP-consuming anabolic pathways.^27^ AMPK is composed of a catalytic (α) subunit and two regulatory (β, γ) subunits. The γ subunit contains two regions that bind energy metabolites (i.e., AMP, ADP, and ATP), in an antagonistic fashion to regulate AMPK activity under energy–stress conditions. Activation of AMPK requires phosphorylation of the α subunit. The activity of AMPK is also controlled by upstream kinases such as liver kinase B1 (LKB1).^28^ Calmodulin-dependent protein kinase kinase-beta (CAMKKβ) is another kinase that activates AMPK under conditions of high cytosolic calcium levels.^29^ AMPK phosphorylates a variety of substrates to alter diverse cellular functions, including fatty acid synthesis/oxidation, glycolysis, mitochondrial biogenesis, and gluconeogenesis. Besides controlling carbohydrate and lipid metabolism, AMPK phosphorylates major mTOR-signaling regulators like TSC2, thus functioning to suppress mTOR signaling that governs anabolic responses, such as protein synthesis. In particular, AMPK signaling in the hypothalamus, a main metabolic tissue in the central nervous system, has been shown to control whole-body energy metabolism (i.e., feeding behavior) by integrating physiologic signals from hormones and nutrients.^30^,^31^

Bang and Kim *et al.* recently demonstrated that IPMK's function in regulating cellular metabolism is not limited to mTOR, but is further extended to hypothalamic AMPK.^32^ Hypothalamic IPMK mRNA and protein levels dynamically respond to energy conditions, such that hypothalamic IPMK levels are downregulated in fasted mice but are markedly elevated upon refeeding. Conditional deletion of the hypothalamic IPMK gene in the arcuate nucleus by adenoviral-mediated introduction of Cre recombinase into the floxed-IPMK gene, in mouse brain, aberrantly increases hypothalamic AMPK signaling activity under *ad libitum* conditions. Similarly, IPMK gene deletion in mouse embryonic fibroblasts produces AMPK signaling defects accompanied by the loss of dynamic regulation of AMPK activity in response to glucose availability.

IPMK appears to be a novel AMPK-binding protein whose binding affinity for AMPK is dynamically controlled by glucose level.^32^ Glucose-starvation in cell culture settings reduces the IPMK-AMPK interaction, which is recovered by glucose treatment. Glucose induces IPMK phosphorylation at tyrosine-174 (IPMK-Y174), which enhances IPMK-AMPK binding and AMPK signaling responses to glucose. Overexpression of dominant-negative IPMK peptides interrupts the binding of IPMK with AMPK and disrupts dynamic AMPK signaling responses to glucose levels. Under energy-stress conditions, the levels of the IPMK-Y174 phosphorylated form are lower, IPMK-AMPK binding is decreased, and AMPK is phosphorylated by LKB1. Glucose stimulation induces phosphorylation of IPMK-Y174 and enhances the interaction between IPMK and AMPK, thereby presumably interrupting LKB1 function and thus modulating AMPK signaling.

A physiological role for hypothalamic IPMK in the control of AMPK signaling and related central energy homeostasis functions was further explored by examining the effects of IPMK deletion within the arcuate nucleus on AMPK signaling and food intake under fasting and refeeding conditions.^33^ Interestingly, compared to controls, the levels of phospho-AMPK signal, as well as the amount of food consumption, are significantly decreased in mice with a specific deletion of the IPMK gene in the arcuate nucleus. These findings strongly suggest that AMPK dephosphorylation and associated anorexic behavior are enhanced in the absence of IPMK. Loss of IPMK in the arcuate nucleus apparently enables AMPK to become a susceptible target for an AMPK-specific protein phosphatase; as a result, AMPK is rapidly inactivated. These data clearly support a new physiological role for IPMK as a key player in the central regulation of hypothalamic AMPK signaling and food intake.

## Conclusions

**Figure 2:**
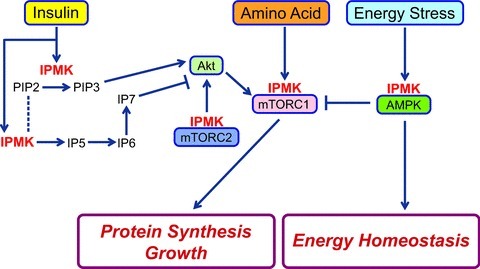
Multiple actions of IPMK in metabolism signaling networks. In response to insulin stimulation, IPMK acts as a PI3 kinase to produce PIP_3_ and thus activate Akt. As an IP kinase, IPMK also generates IP_4_ and IP_5_, precursors of inositol poly- and pyrophosphates. IP6K1-dependent 5-IP_7_ inhibits Akt activation, thereby negatively regulating downstream insulin actions such as adipogenesis. Acting in a catalytic activity-independent manner, IPMK binds, stabilizes, and mediates the activation of mTORC1 in response to amino acids. The kinase activity of IPMK appears to mediate insulin-dependent mTORC2 activation, but the detailed molecular mechanisms remain unclear. Another IPMK signaling target is AMPK, which governs central metabolic homeostasis by controlling food intake. The binding of IPMK to AMPK appears to be the key event in the modulation of AMPK signaling by upstream AMPK kinases or protein phosphatases.

A growing body of evidence suggests the functional significance of IPMK in the regulation of metabolism signaling networks in response to hormones (e.g., insulin), amino acids, and energy levels (Fig. 2). Despite substantial advances in understanding the physiological functions of IPMK, many questions regarding the mechanistic details of IPMK actions remain unanswered. For example, what stoichiometric relationships between IPMK and its multiple signaling targets (e.g., mTOR and AMPK) are possible? And what is the glucose-regulated protein tyrosine kinase that mediates IPMK phosphorylation and associated IPMK signaling?

Importantly, IPMK is directly or indirectly involved in other major cellular events, such as gene expression and apoptosis. In conjunction with IP6K, the IP kinase activity of IPMK plays a critical role in generating IP_7_ (Fig. 1), which is either an Akt inhibitor or a regulator of p53-mediated apoptosis, depending on cellular context.^34^,^35^ On the other hand, the PI3 kinase activity of IPMK, acting in concert with the classical p85/p110-PI3K, is responsible for growth factor-stimulation of Akt signaling.^14^ Future studies on how the PI3 kinase, IP kinase, and/or kinase activity-independent signaling functions of IPMK can be switched on or off may reveal a crucial mechanism of IPMK regulation. Presumably, the two major catalytic roles of IPMK work coordinately with the noncatalytic actions of IPMK in mediating responses of mTORC1 to amino acids.

Another major area of future IPMK research is to identify novel IPMK functions in metabolism or related signaling pathways. Recent reports on an unexpected role for IP_4_, one of the products of IPMK, in the assembly and activation of histone deacetylase complexes clearly suggests the presence of additional targets of IPMK-mediated epigenetic signaling in mammalian cells.^36^ The nucleocytoplasmic shuttling of IPMK and the identification of the nuclear receptor steroidogenic factor 1 as a new IPMK target also indicate the complexity of IPMK signaling actions.^37^,^38^ Studies of tissue- or cell-type–specific IPMK functions using conditional gene knockout strategies may help to elucidate the physiological roles of IPMK *in vivo*. Because IPMK regulates Akt and mTOR in cell culture settings, we speculate that IPMK-null organs in mouse model systems may exhibit attenuated insulin responses and related metabolic changes affecting glucose homeostasis and adipogenesis. IPMK-mediated AMPK signaling also provides another potential regulatory layer critical for allowing target tissues and the whole body to accurately gauge and adapt energy and nutrient levels. The question of how multiple IPMK signaling functionalities integrate different types of upstream metabolic stimuli warrants further investigation. Accordingly, drugs that perturb IPMK signaling actions on major metabolic signaling targets, such as Akt, mTOR, and AMPK, may have therapeutic applications for metabolic syndromes (e.g., obesity, type 2 diabetes) and related eating disorders, such as bulimia and anorexia nervosa.
